# Our New York Letter

**Published:** 1891-06

**Authors:** 


### OUR NEW YORK LETTER.

NEW YORK, May, 17, 1891.

The new law regulating the standard of medical education in this State will prove a death blow to a certain percentage of those colleges that have been up to this time deriving a lucrative business from the sale of medical diplomas. For the future, a good many of these diplomas will count for nought, unless the holders of them are capable of passing the required examination before the Board of Regents in Albany, and the applicant depositing, in addition thereto, the sum of thirty-five dollars into the treasury of the University of the State of New York before he is given an order for examination by the Board of Medical Examiners; the Regents having the power to revoke any such license issued by them, on satisfactory evidence of infamous conduct, or malpractice on the part of the physician to whom it has been issued.

The examinations shall be both written and oral, and the subjects for examination shall be:

1. Anatomy.
2. Physiology and medical chemistry.
3. Pathology.
4. Surgery.
5. Obstetrics, and diseases of women and children.
6. Hygiene.
7. Toxicolog yor pharmacology.

Any medical college which fails to give adequate instructions in all of these subjects, shall be excluded by the Board of Regents from recognition as qualifying its graduates for their license.

This law, it can be readily understood has been the one thing wanted to force these recreant medical colleges to come into line, and advance the standard of medical education in this city and State.

The next thing in order will be the sudden and unpected demise of a few of the so-called medical colleges in this vicinity ; and aside from the teachers of these institutions, this consummation will be mourned by but few practicing physicians of New York.

Dr. Herman Collyer, of the New York Polyclinic, recently re-the ported the birth of a viable child, at the sixth month, before section of Gynecology of the New York Academy of Medicine. The mother was 33 years old, and had been married eleven years, having four children living all born at full term, and one miscarriage. On February 4th of this year delivered of a small child weighing two pounds and two ounces. The testes of the infant had not descended at the time of birth, and the finger nails were just making their appearance. The child is continuing to thrive in every respect.

Dr. R. W. Taylor, who succeeded Dr. Fessenden N. Otis, as Professor of Genito-Urinary Diseases at the College of Physicians and Surgeons, of this city, in a recent lecture on the treatment of syphilis says, that there is no drug which in his experience has been so efficacious during the early stages of syphilis as the protoiodide of mercury, but there is a limit to the use of this remedy. It is advisable to give it for the first month or two, and then to use inunctions of the mercurial oitment. The blue pill in the treatment of this affection he considers the most uncertain and capricious remedy that could be employed. When pushed it will produce salivation as rapidly as possible, but when it is acting blandly, it is doing no good at all. In case inunctions should not agree with the patient, he says he has found a sovereign remedy in the injections of the bichloride of mercury. He has been employing this method for over twenty-three years, and he has secured the very best results from the use of these injections. Speaking of the curability of syphilis he says, the keynote to the treatment of this affection is in pushing the mercurials during the first year of the disease, and that he can look back time and again and see children grow up to puberty vigorous and healthy, both mentally and physically, whose fathers he had at one time treated for syphilis. The reason why some patients remained uncured of this affection, is because of the difficulty of keeping them under observation and treatment for the required length of time, which is at least two years.

Dr. Delafield, at a recent clinic at the College of Physicians and Surgeons, stated that he was called to see a patient with the following history:

He was a man 35 years of age, of somewhat intemperate habits, who sustained, while under the influence of liquor a traumatism over the region of the liver. This did not trouble him at first, but in a day or so he developed pain over the liver, had fever, and felt sick enough to go bed. He remained in bed, the pain and fever continuing, and then began to waste gradually away. After suffering in this manner for some weeks, it was noticed that the liver was apparently enlarged. When Dr. Delafield saw the patient he was constantly vomiting, and had the peculiar expression characteristic of septic troubles. The attending physician suspected abscess of the liver, and on aspiration, drew out no pus. The upper border of the liver was a little higher than normal, while its lower border, begining on the left side extending down symmetrically into the abdominal cavity, as far as the umbilicus. Not only was the liver enlarged in a downward direction, but also forward so that the anterior abdominal wall was pushed up, all the characteristic outlines of an enlarged liver being present. The attending physician made a diagnosis of amyloid degeneration of the liver, but Dr. Delafield thought the man had either a suppurative process of the liver, or a rapid new growth of that organ, though the clinical appearances were strongly suggestive of suppuration. The man was so sick at this time he did not deem it wise to make a second aspiration to verify this presumptive diagnosis of the existence of pus in the liver. That morning he received a note from the attending physician saying that the man had died, that the liver had been examined, and he had found something that did not enter into his calculations in the matter of diagnosis. The tumor was an enlarged gall bladder that had been spread out under the lower surface of the liver so as to form a symmetrical and continuous tumor with the latter. The gall bladder was found distended with pus. There was no cause for this condition but the blow which the man received over the region of the liver.

Dr. R. M. C. Page, of the New York Polyclinic, speaking of the hemorrhage of phthisis says, that ergotine and morphia, given internally, are the only two remedies that can safely be relied upon in this emergency. He lately saw a patient at Brompton Hospital, England, where every remedy known to medicine was used, but in vain, to stop an alarmingly profuse hemorrhage in a phthisical patient, and the physicians had finally to have recouse to these drugs given hypodermically, which at once arrrested the hemorrhage and quieted the fears of the patient. For the night sweats of phthisis, he recommends the following as the most efficient and prompt remedy;

℞ Pul. zinc oxid., gr. xii.

S. To be taked at bed time.

He has used this remedy, he says, in every case of night sweats that he has treated of late years, and have never known it to fail in producing a very beneficial effect.

Dr. John A. Wyeth, deprecates the employment of injections of any kind in gonorrhœa, and says that they generally do more harm than good. He recommends the use of the oil of gaulthesia, in six drops doses three times a day, as a more efficient and safer remedy for acute urethritis than any other preparation generally advised for that purpose. The common practice of placing a piece of lint or absorbent cotton over the meatus is wrong in practice, as it interferes with the free drainage, and brings the acrid discharges from the urethra in direct contact with the glans and prepuce. A bag of oiled silk or an ordinary cloth should be made large enough to fit loosely over the penis, and held in place by strings which pass up to a belt worn around the waist. P. J. R.

---

DIAGNOSIS BETWEEN INTRA AND EXTRA CAPSULAR FRACTURES OF THE FEMUR.—Prof. Keen, of Philadelphia, gives the following differential diagnosis between these fractures:

Intra-Capsular: 1. Slight injury. 2. Slight Contusion. 3. Shortening increases. 4. Feeble crepitus. 5. Leg nearly helpless. 6. Shorter radius of rotation. 7. Pain moderate. 8. Usually occurs in persons over fifty years of age. 9. In women as a rule.

Extra-Capsular: 1. Severe injury. 2. Usually severe contusion. 3. Does not increase. 4. Distinct crepitus. 5. Absolutely so. 6. Still shorter radius of rotation. 7. Pain severe. 8. Usually occurs in persons under fifty years. 9. Generally occurring in men.—*Times and Register.*

